# Chaos theory discloses triggers and drivers of plankton dynamics in stable environment

**DOI:** 10.1038/s41598-019-56851-8

**Published:** 2019-12-30

**Authors:** Irena V. Telesh, Hendrik Schubert, Klaus D. Joehnk, Reinhard Heerkloss, Rhena Schumann, Martin Feike, Arne Schoor, Sergei O. Skarlato

**Affiliations:** 1 Zoological Institute, Russian Academy of Sciences, St. Petersburg, 199034 Russia; 2 Institute of Cytology, Russian Academy of Sciences, St. Petersburg, 194064 Russia; 3 University of Rostock, Institute of Biosciences, Rostock, 18059 Germany; 4 CSIRO Land and Water, Black Mountain, Canberra, ACT 2601 Australia; 5 University of Rostock, Biosciences, Subject Didactics Biology, Rostock, 18051 Germany

**Keywords:** Food webs, Population dynamics, Theoretical ecology

## Abstract

Despite the enticing discoveries of chaos in nature, triggers and drivers of this phenomenon remain a classical enigma which needs irrefutable empirical evidence. Here we analyze results of the yearlong replicated mesocosm experiment with multi-species plankton community that allowed revealing signs of chaos at different trophic levels in strictly controlled abiotic environment. In mesocosms without external stressors, we observed the “paradox of chaos” when biotic interactions (internal drivers) were acting as generators of internal abiotic triggers of complex plankton dynamics. Chaos was registered as episodes that vanished unpredictably or were substituted by complex behaviour of other candidates when longer time series were considered. Remarkably, episodes of chaos were detected even in the most abiotically stable conditions. We developed the Integral Chaos Indicator to validate the results of the Lyapunov exponent analysis. These findings are essential for modelling and forecasting behaviour of a variety of natural and other global systems.

## Introduction

Chaotic dynamics is a common feature for biological, physical, chemical, social and other global systems although their complex behaviour is displayed at very different time scales within highly variable spatial formats. Due to low predictability, chaotic dynamics are not easily reconciled with the conventional cause-effect relationships, concepts and paradigms that contribute to our understanding of systems’ functioning^1–3^. For example, urbanistics and architecture^4^, peacebuilding processes^5^, conflict resolution theories and community development practices^6^, as well as manifestation of democracy as a societal model that represents myriads of related individuals and social groups with conflicting viewpoints, ideas and demands^7^ generate interactions with wildly oscillating effects. The processes in natural biological systems are likewise complex, nonlinear and dynamical, and their outcomes can demonstrate chaotic behaviour^8–11^. Meanwhile, mechanisms behind those peculiar patterns are not fully understood. This hampers modelling of natural ecosystems’ vulnerability to external stresses like harmful algal blooms or alien species invasions, and precludes their management, particularly in the economically significant highly populated coastal ecosystems worldwide^12,13^.

Mathematical models predict that depending on strength of regulatory mechanisms interactions between species can generate very complicated dynamics that have high sensitivity to the initial conditions – the so-called ‘butterfly effect’^14^, which was described in the chaos theory^15–20^. Consequently, along with stable equilibria and cyclic dynamics, populations of living organisms can show unpredictable chaotic and fractal behaviour^11,21–23^. Chaotic behaviour was coined ‘divergence of nearby trajectories’ which is a hallmark of chaos also referred to as ‘complex behaviour’^24^.

Experimental demonstrations of chaotic behaviour in ecology are scarce and have been mostly confined to relatively simple laboratory systems, such as cultures of flour beetles^25^, a community of three species in chemostat cultures^26,27^, two mutualistic microbial guilds^28^, or phytoplankton assemblages in the incubators^24^ and microcosm experiments with natural estuarine plankton subjected to different disturbances^29^. Specifically, Roelke and co-authors^24^ used numerical models and determined that the phytoplankton may be structured in such a way that allows complex behaviour to arise in the microcosms receiving moderate or low disturbances; meanwhile, complex behaviour may be suppressed by large disturbances^24,29^.

Originating from theoretical ecology^30^, the debate about chaotic behaviour of natural communities has intensified during the recent decade since the signs of chaos were reported in the larger, long-term experimental system with natural populations^8^ and in the lake-scale ecosystems^10^. These studies targeted plankton communities because due to short life cycles of the organisms they provide time series that are sufficiently long to allow for community dynamics analysis^31^. Moreover, plankton dynamics is rather well studied^32^, although it is conventionally interpreted using the Plankton Ecology Group (PEG) model to describe the interplay between strong external triggers setting the frame for internal biotic interactions as drivers^24,33^.

## Can There Be Chaos in A System Without External Triggers?

For many ecologists the question what is chaos – mathematical artefact or ecological reality^30^ – is still open, irrespective of the examples in nature reported so far. Eventually, the mechanistic models^34–36^ have brought the effect of abiotic factors back into fashion through seasonal forcing of vital rates^37^. For example, the vulnerability of small populations to environmental changes is a phenomenon, which is of major concern in conservation biology; however, the resulting dynamics of those populations was shown to be triggered by external factors^38^. Thus, it is an open question whether or not the observed complex population behaviour is just reflecting the chaotic patterns of the external triggers.

Some of the uncertainties were resolved in the experiment reported by Benincà with co-authors^8^, who presented the exceptionally long (2,319 days) plankton time series sampled with high frequency. However, although this experiment was accurately designed, skilfully implemented and carefully analyzed, still it left some important questions unanswered. One of the main points of concern is the lack of replicates in this experiment; another open question is instability of external factors acting as triggers for plankton dynamics.

External triggers, especially irradiance and temperature, are responsible for setting the frame within which the biological interactions take place in nature^39^. In the experimental mesocosms, the organisms also have to tolerate fluctuations of the external factors, and the specific tolerance ranges correlate mainly with longevity. Therefore, modification of the abiotic environment is followed not only by changes in the abundance of species but also by species composition shifts^24,29^. The latter is most pronounced for short-lived unicellular organisms compared to larger, multicellular organisms^40,41^. However, the question whether or not chaos can be detected in the systems with stable abiotic parameters remains unresolved.

Here we demonstrated the interplay of triggers and drivers of plankton dynamics in multi-species communities under artificially stabilized abiotic conditions in the replicated yearlong mesocosm experiment. We set forward and tested two research hypotheses (RHs). According to the RH1, the natural phenomenon of the “paradox of chaos” implies that biotic interactions act as both triggers and drivers for plankton dynamics in the systems lacking abiotic variability. The RH2 suggests that complex behaviour is an option which can be expressed just in episodes at any level of biological organization. If chaos is an inherent character of the systems, complex behaviour should always appear when the observation time span is sufficiently long. The question whether such chaotic behaviour is a mandatory feature of the systems lacking external triggers or just an episode/probability had neither been tested experimentally nor proved by previous observations.

**Figure 1:**
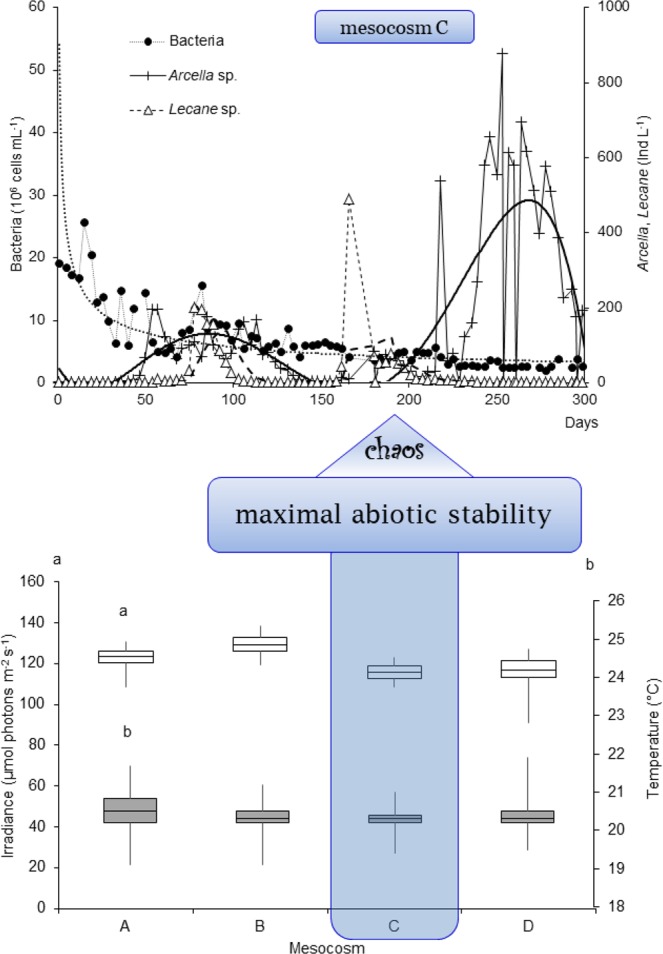
Chaotic dynamics revealed in plankton at maximal abiotic stability. *Lower panel*: Box-Whisker plots of irradiance (a) and water temperature (b) in mesocosms (A–D) (number of observations *n* = 130 in each mesocosm). *Upper panel*: Abundance dynamics of bacteria (approximated by power function: y = 54.013x^−0.486^, R² = 0.669, *n* = 97), testate amoebae *Arcella* sp. (polynomial function: y = −2E-08x^5^ + 2E-05x^4^−0.0035x^3^ + 0.3077x^2^ − 8.0599x + 45.17, R² = 0.627, *n* = 97), and rotifers *Lecane* sp. (*n* = 97, moving average with a period *n* = 6) in the mesocosm C, which was characterized by the highest abiotic stability. Chaotic behaviour of bacteria, protozoans and rotifers was detected by Lyapunov exponent analysis and verified by the Integral Chaos Indicator (see text for explanations).

To test these hypotheses, we designed a set of four mesocosms with identically stable abiotic conditions^42^ and investigated diversity, abundance and interactions of major planktonic components (uni- and multicellular as well as colonial representatives) from different trophic levels (primary producers, herbivorous and carnivorous consumers, and decomposers; top predators were lacking in the system) during a yearlong experiment. The experimental plankton community developed from an inoculum of the Baltic Sea water and included bacteria, picocyanobacteria, phytoplankton, micro- and mesozooplankton (see Methods). Plankton was cultured under constantly controlled abiotic conditions to exclude extrinsic influences^42^. We detected episodes of chaotic plankton dynamics in all mesocosms; the number of these episodes was the highest in the most abiotically stable environment (Fig. 1).

## Controlled Abiotic Stability

The initial abiotic conditions in four mesocosms (labelled A to D) were very similar or even identical, providing high degree of long-term stability of the major external triggers of plankton dynamics (irradiance, temperature and salinity), which was maintained in all replicate mesocosms throughout the experiment (Fig. 1, lower panel; Supplementary Fig. S1). Concentration of nutrients – soluble reactive phosphorus (SRP), nitrate, nitrite, ammonium, total nitrogen (TN) and total phosphorus (TP) in seston and their dynamics were largely similar in all replicate mesocosms, with only minor exceptions and without any distinct differences in trends and variation ranges of the parameters (Supplementary Fig. S2). These patterns of nutrient dynamics, although not always clearly visible in the graphs based on the initial non-transformed data, were proved to be statistically similar by the Spearman Rank Correlation Analysis (Supplementary Table S1). For dissolved inorganic nitrogen (DIN) concentrations, all Pearson correlation coefficients (*p*-values) exceeded 0.2, most of them being > 0.5; this witnesses for very high similarity of patterns in all mesocosms. Patterns of PO_4_^3−^ (dissolved inorganic phosphorus, DIP) were very similar in A, B and C (*p* > 0.2), while this similarity for C/D was the lowest. The *p*-values for SRP in A/D and B/D were the lowest (Supplementary Table S1, numbers in bold), suggesting that mesocosm D was different from the others in terms of PO_4_^3−^ dynamics (Supplementary Fig. S2e).

## Trends in Plankton Dynamics

Plankton composition, abundance, and their dynamics varied in time and strength demonstrating comparable trends. Dynamics of micro-biotic components (nutrients concentration, the bacteria and cyanobacteria^43^) was relatively uniform (Supplementary Fig. S2a–g). Microzooplankton – protozoa (the testate amoeba *Arcella* sp.) and rotifers (*Lecane* sp., *Colurella* sp., *Filinia longiseta*, *Brachionus quadridentatus* and *Keratella cochlearis*) demonstrated patterns of annual population dynamics that varied in different mesocosms (Supplementary Fig. S2h–m). Abundance of mesozooplankton (cladocerans *Alona* sp., copepods *Eurytemora affinis*, *Acartia tonsa*, juvenile cyclopoid copepods and other zooplankters) were irregular, representing natural cycles of population development against the background of the available food resources (Supplementary Fig. S3a–e). The zooplankton species composition varied substantially between the replicate mesocosms; however, the overall zooplankton biomass had generally similar temporal dynamics, whereas the magnitude of peaks and dips differed significantly (Supplementary Fig. S3f–l). Thus, the individual kinetics of plankton components demonstrated differences between mesocosms, mainly with respect to zooplankton composition.

**Figure 2:**
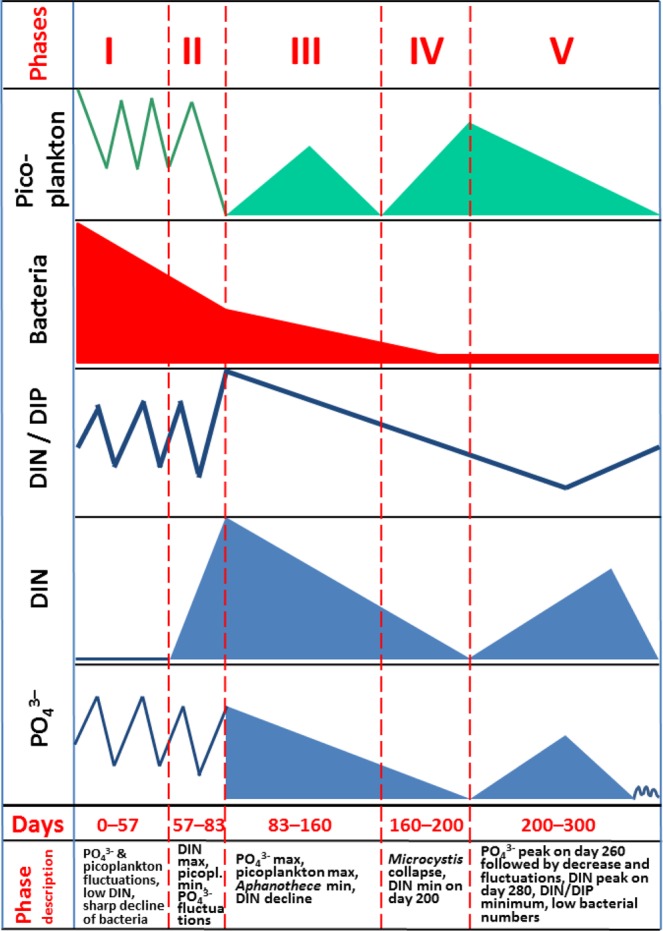
Phases in plankton dynamics: trends, duration and description. Scheme based on the kinetics of picoplankton, bacteria, DIN and PO_4_^3–^ concentrations, and DIN/DIP ratio (for the background data see Supplementary Fig. S2).

Despite the varying community structure, five phases in plankton dynamics can be distinguished in all experimental mesocosms, as demonstrated by the scheme based on kinetics of picoplankton, bacteria, DIN and DIP concentrations, and DIN/DIP ratio (Fig. 2). Those phases were evident in all mesocosms, and this is a proof that our mesocosms were true replicates and plankton therein behaved similarly with respect to periodicity.

**Figure 3:**
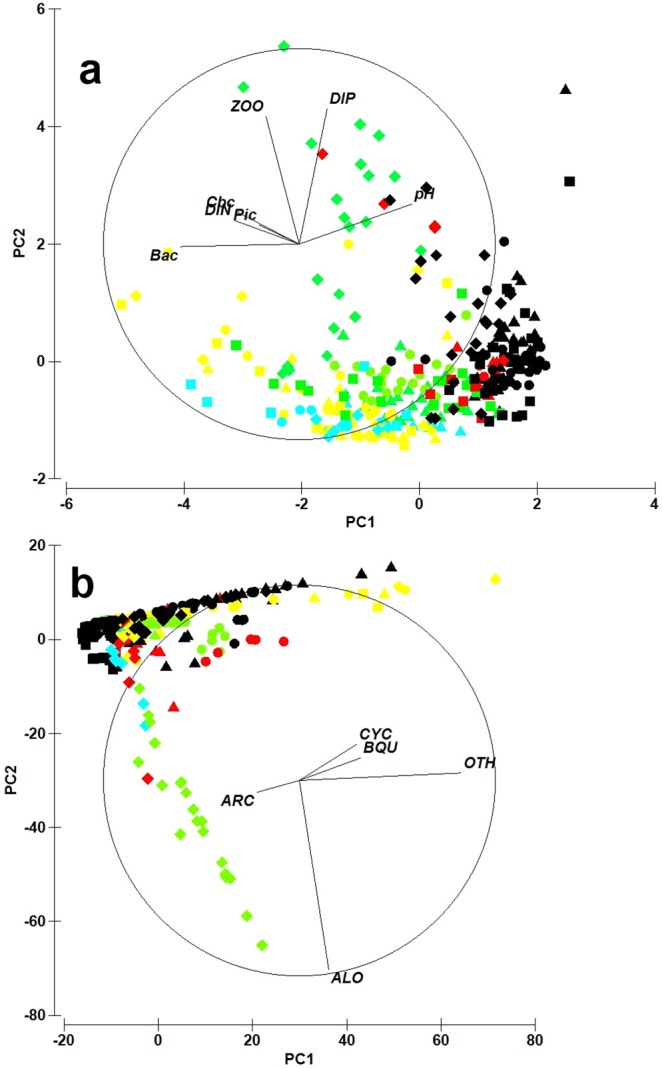
Results of PCA analysis of plankton dynamics and abiotic parameters in mesocosms. (**a**) Analysis based on zooplankton biomass and the overall abundance of Cyanobacteria, summing up all species counted. (**b**) Analysis based on species composition and abundance of zooplankton and Cyanobacteria. Vectors are shown only for the correlation coefficients *r* > 0.2. Symbols indicate mesocosms: triangle – A, circle – B, square – C, diamond – D. Colour codes for phases I–V of plankton development: yellow – I, blue – II, green – III, red – IV, black – V (for description of phases see Fig. 2). Abbreviations: ALO – *Alona* sp., ARC – *Arcella* sp., Bac – bacteria, BQU – *Brachionus quadridentatus*, Cbc – cyanobacteria colonies, CYC – *Cyclotella* sp., DIN – dissolved inorganic nitrogen, DIP – dissolved inorganic phosphorus, OTH – other zooplankton, Pic – picocyanobacteria, ZOO – zooplankton biomass.

The principal component analysis (PCA) of the overall zooplankton biomass versus a set of abiotic characteristics and microplankton data allowed revealing a specific pattern (Fig. 3). This pattern could be explained neither by any single factor nor by their combination: eigenvalues were low and the percentage of variability explained by them was negligible (Supplementary Table S2). Reduction of the dataset by excluding the first period of ca. 50 days (the ‘acclimation phase’) did not change the picture substantially enough, and the pairwise test for differences by ANOSIM resulted in very low R-values ranging from 0.008 (in mesocosms B, A and A, C) to 0.012 (in B, D and C, D). The bacterial abundance dominated the first component with a loading of −0.592 (Supplementary Table S3). Additional test using a nested two-way approach failed detecting any significant differences between mesocosms as well as between the individual phases within each mesocosm (Supplementary Table S4).

Results of the PCA analysis showed that in both cases (when zooplankton taxonomic composition was taken into account, and when zooplankton was considered as a trophic level of primary consumers irrespective of the species composition), no statistical differences between mesocosms were registered (with only one exception, see Supplementary Fig. S3b).

## The Integral Chaos Indicator

Chaotic behaviour of the biotic parameters in the experiment was detected by the Lyapunov exponent (Ly) analysis. Results are presented for the stationary window time series and for all available time series (Supplementary Figs. S3 and S4, respectively). The histograms and probabilities of the fitted slope of Ly-values are shown for 100 randomly shuffled time series of the stationary window dataset (Supplementary Fig. S5).

**Figure 4:**
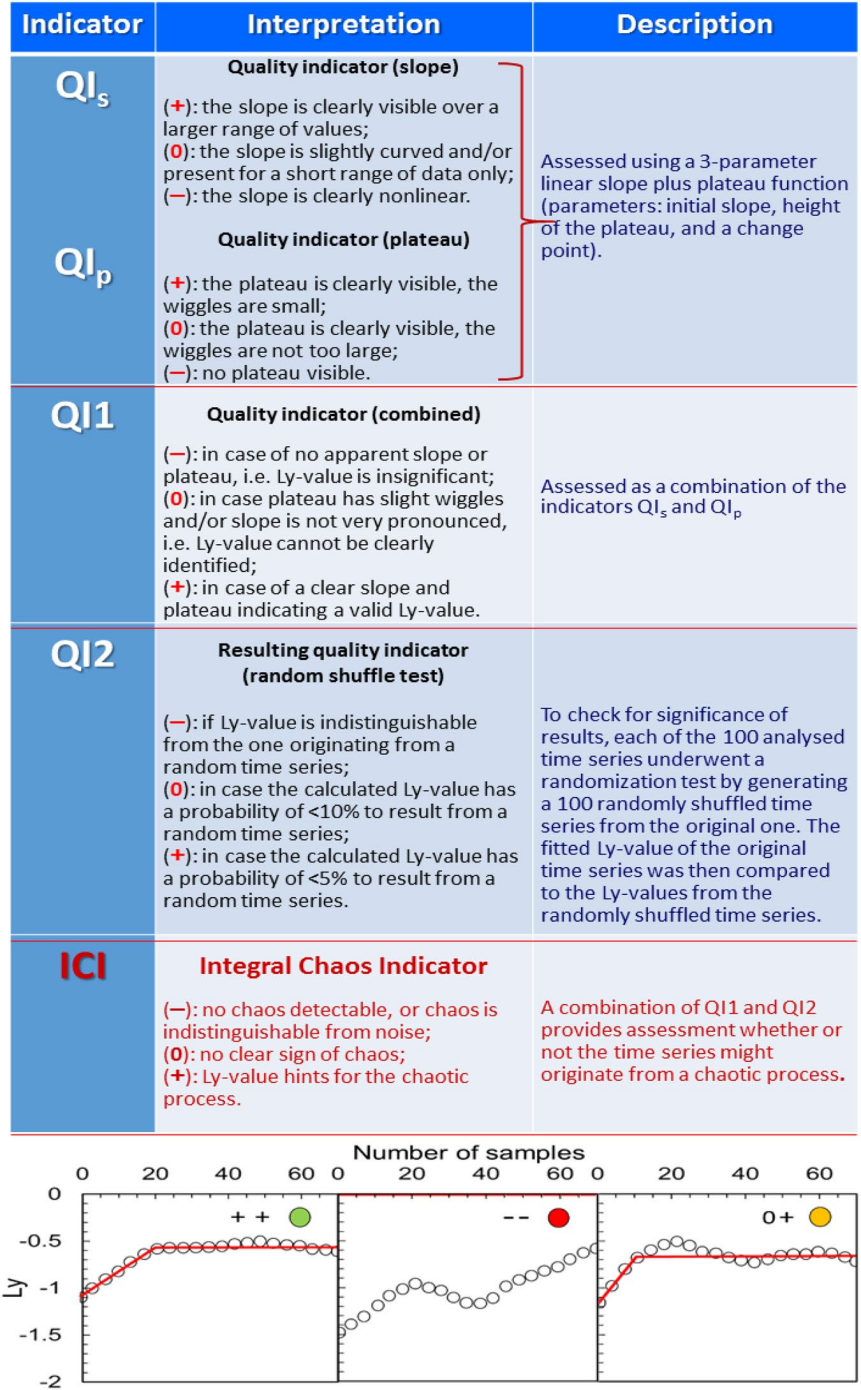
Description of the quality Indicators QI_s_, QI_p_, QI1, QI2 and ICI, and visualization of assessment of the Lyapunov exponents using these indicators for the stationary window time series. Each graph in the lower panel shows the points calculated by Tisean v3.0.1 package (empty circles), a combined fit for the linear increase and plateau (red line), quality assessment (+, 0, −) by the indicators QI1 (first symbol) and QI2 (second symbol), and a large colour dot indicating the relevance (reliability) of the calculated Ly-values: green – positive test for both quality indicators (QI1 and QI2), yellow – positive test for just one of the indicators, red – negative tests for both indicators.

For verification of the calculated Ly-values, a set of indicators was developed (Fig. 4). The output of the analysis was fitted using a 3-parameter linear slope plus plateau function and then assessed using the combined linear-plateau fit, a set of quality indicators (QI_s_, QI_p_, QI1, QI2), and the original Integral Chaos Indicator (ICI). The ICI was calculated based on the assumption that there was no hint for chaotic time series in all those cases when QI1 or QI2 or both provided negative quality assessment of Ly-values (−), i.e. no slope was apparent, or the slope was indistinguishable from noise. A sign of chaotic processes (+) was only considered in those cases when both QI1 and QI2 were positive.

The values for the fitted Lyapunov exponent, the standard deviation of the fitted slope, the indicators QI1, QI2 and ICI are given in Table 1. The results proved that nearly all Ly calculations, including the deterministic test time series, produced positive Ly-values that are signs of complex behaviour. However, testing these results by the indicator QI1 showed that the false positive Ly-values were in many cases as frequent as expected for the ‘true’ positives. Meanwhile, the random shuffle test and the indicator QI2 allowed eradicating most of the false positives by showing that their values were not distinguishable from those generated by the random time series (Table 1). Nevertheless, signs of potential chaotic behaviour were still confirmed statistically for some biotic components: testate amoebae *Arcella* and rotifers *Lecane*; surprisingly, such tendencies were not revealed for bacteria, picophytoplankton and SRP (Table 1).

**Table 1:** Values of the Lyapunov exponent and their assessment by quality indicators QI1, QI2, and the Integral Chaos Indicator.

Parameter	Ly^*^	±std	QI1	QI2	ICI	Parameter	Ly	±std	QI1	QI2	ICI
Stationary window						All available dates					
**Aarc**	**0.05833**	**0.00623**	**+**	**+**	**+**	**Aarc**	**0.0594**	**0.01172**	**0**	**+**	**0**
*Barc*	*0.03962*	*0.00681*	−	−		Barc	0.04382	0.00326	**+**	−	
**Carc**	**0.02985**	**0.00416**	**+**	**+**	**+**	Carc	0.04153	0.00183	**+**	−	
Darc	0.04478	0.00521	**+**	−		Darc	0.04274	0.00617	0	−	
**Alec**	**0.03566**	**0.00156**	**+**	**0**	**0**	**Alec**	**0.05339**	**0.00163**	**+**	**+**	**+**
**Blec**	**0.05098**	**0.00745**	**+**	**+**	**+**	Blec	0.04921	0.00407	0	0	0
**Clec**	**0.02432**	**0.0042**	**+**	**+**	**+**	Clec	0.03973	0.00374	−	0	
Dlec	0.01974	0.0017	−	**+**		**Dlec**	**0.05765**	**0.00332**	**+**	**+**	**+**
Ason	0.02879	0.0102	0	−		Ason	0.04238	0.00519	**+**	−	
Bson	0.04362	0.00386	0	**+**	0	Bson	0.03389	0.0026	**+**	−	
*Cson*	*0.04284*	*0.03337*	−	−		Cson	0.04264	0.00459	**+**	−	
Dson	0.01616	0.00142	−	**+**		Dson	0.02183	9.6E-4	−	**+**	
Abac	0.03841	0.01894	0	−		**Abac**	**0.03015**	**0.00774**	**0**	**+**	**0**
Bbac	0.03359	0.0056	0	−		Bbac	0.03918	0.00329	**+**	−	
**Cbac**	**0.04805**	**0.0025**	**+**	**+**	**+**	Cbac	0.04177	0.00564	**+**	−	
Dbac	0.03318	0.01312	0	−		**Dbac**	**0.0252**	**0.00263**	**0**	**+**	**0**
Apic	0.02648	0.00211	**+**	0	0	Apic	0.03401	0.00263	**+**	−	
*Bpic*	−	−	−	−		Bpic	0.05094	0.00893	**+**	0	0
Cpic	0.04187	0.00361	**+**	−		Cpic	0.03818	0.00547	**+**	−	
Dpic	0.05326	0.01658	0	**+**	0	**Dpic**	**0.05818**	**0.00627**	**0**	**+**	**0**
ASRP	0.0246	0.00433	0	−		**ASRP**	**0.02505**	**0.00485**	**0**	**+**	**0**
BSRP	0.04122	0.02393	0	−		BSRP	0.04887	0.02819	−	0	
CSRP	−0.00432	0	−	**+**		CSRP	0.02832	0.01527	−	**+**	
**DSRP**	**0.06932**	**0.02413**	**0**	**+**	**0**	DSRP	0.07263	0.04868	−	**+**	
Aext	0.02911	0.00238	0	0	0	Aext	0.04494	0.00326	**+**	−	
**Bext**	**0.02121**	**5.4E-4**	**+**	**+**	**+**	**Bext**	**0.04713**	**0.00273**	**+**	**0**	**0**
Cext	0.03687	0.00527	**+**	−		**Cext**	**0.05613**	**0.00426**	**+**	**+**	**+**
Dext	0.03424	0.00328	0	−		**Dext**	**0.02951**	**0.00173**	**+**	**+**	**+**
Aph	0.04481	0.00319	**+**	−		Aph	0.04403	0.00282	0	−	
Bph	0.03766	0.01383	0	−		**Bph**	**0.05002**	**0.00956**	**+**	**0**	**0**
**Cph**	**0.04603**	**0.00427**	**+**	**+**	**+**	Cph	0.04211	0.00154	**+**	−	
Dph	0.04389	0.00465	**+**	−		Dph	0.04117	0.00448	**+**	−	
**Atem**	**0.04221**	**0.00195**	**+**	**0**	**0**	Atem	0.04809	0.00359	0	0	0
Btem	0.04189	0.00418	**+**	−		**Btem**	**0.04923**	**0.00619**	**+**	**+**	**+**
Ctem	0.03906	0.00299	**+**	−		**Ctem**	**0.05136**	**0.00326**	**0**	**+**	**0**
**Dtem**	**0.05047**	**0.00444**	**+**	**0**	**0**	Dtem	0.03688	0.00256	**+**	−	
Airr	0.0421	0.00757	0	−		Airr	0.0481	0.00451	0	−	
Birr	0.04857	0.01005	−	**+**		Birr	0.04571	0.00592	0	−	
Cirr	0.04093	0.00404	**+**	−		**Cirr**	**0.04555**	**0.00636**	**0**	**+**	**0**
**Dirr**	**0.02853**	**0.00609**	**0**	**+**	**0**	**Dirr**	**0.03293**	**0.00558**	**0**	**+**	**0**
**Asal**	**0.09417**	**0.00945**	**0**	**+**	**0**	**Asal**	**0.06935**	**0.00587**	**0**	**+**	**0**
Bsal	−0.01226	0	−	**+**		Bsal	−0.02437	0	−	**+**	
*Csal*	*0.04035*	*0.00469*	−	−		*Csal*	*0.04102*	*0.00353*	−	−	
Dsal	0.10432	0.02823	−	**+**		Dsal	0.07965	0.01841	−	**+**	−
test1	0.01466	0.0017	−	**+**		**test1**	**0.01851**	**0.00425**	**0**	**+**	**0**
**test2**	**0.01879**	**0.00163**	**0**	**+**	**0**	**test2**	**0.02971**	**0.00228**	**0**	**+**	**0**
*test3*	*0.0364*	*0.00608*	−	−		test3	0.06913	0.00867	−	**+**	
test4	−	−	−	**+**		test4	−	−	−	**+**	
test5	0.01791	5E-4	−	**+**		test5	0.02457	6.8E-4	−	**+**	

*Lyapunov exponent (Ly), standard deviation of fit (±std), indicators for the quality of fit (QI1) and the random shuffle test (QI2), and the Integral Chaos Indicator (ICI), calculated for the stationary window (left-side part) and all available dates (right-side part). The capital letters in front of the parameter abbreviations indicate the respective mesocosms (A, B, C and D). Abbreviations: arc – *Arcella* sp., lec – *Lecane* sp., son – other zooplankton, bac – bacteria, pic – picophytoplankton, SRP – soluble reactive phosphorus, ext – light extinction, ph – pH, tem – temperature, irr – irradiance, sal – salinity; test1: deterministic, test2: test1 + random, test3: random, test4: ordered Aarc, test5: truncated Lorenz attractor, quasi-deterministic;–− negative test results. For explanation of the results (+, 0 and −) see text and legend to Fig. 4. Relevance of the calculated Ly-values tested by QI1, QI2 and ICI is indicated by bold font for all parameters tested positive for at least one of the quality indicators (QI1 and QI2) without contraindication with the respective second one. The cases of negative test results for both indicators are given in *italic* font.

For the stationary window data, the assessment allowed revealing 5 biotic parameters with reliable signs of complex behaviour: *Arcella* in mesocosms A and C, *Lecane* in B and C, and bacteria in C. In addition, the abiotic parameters – light extinction in B, and pH in C – also demonstrated positive ICI results that indicate chaotic behaviour.

When the entire dataset was analyzed, including the data from the ‘noisy’ acclimation phase, none of the former candidates from the stationary-window dataset expressed the reliable complex behaviour, although now they were exhibiting higher Ly-values compared to the previous assessment (Table 1). Moreover, in this case some other candidates were exposing signs of chaos since the data from the acclimation phase added extra variability (Table 1).

Variation of the reliably positive Ly-values was high, ranging from 0.021 to 0.058 per 3.5 days. Low Ly-value (0.021) for light extinction in mesocosm B was quite close to the values generated for the deterministic or only slightly random test cases 1, 2 and 5 (Table 1). Relatively frequent positive results of the random shuffle test for salinity and the test cases assessed by QI2 suggested that those Ly-values most likely originated from a random noise process. The latter conclusion was also supported by a lack of clear slope/plateau as indicated by QI1 (Table 1). The truncated Lorenz time series (test 5) was classified as non-chaotic showing no plateau; the information contained in this short snippet of the chaotic Lorenz attractor was not sufficient to detect a clear sign of chaos.

Calculation of the Ly-values provided remarkable results since initially quite a number of biotic parameters exhibited positive Ly-exponents with rather low standard deviations (Table 1). Evaluation of these results for probability of being false-positive by comparing them with the test time series provided the unexpected outcome. Specifically, for the stationary window dataset, five biotic parameters exhibited signs of complex behaviour; three of those were discovered in one mesocosm (C). Among those parameters, at least 3 components of the food web and pH – a feature strongly influenced by the biotic activity – showed signs of chaotic behaviour. However, these signs vanished when the entire dataset was analyzed. Thus, surprisingly, addition of the strongly variable data from the initial acclimation phase that were characterised by the most drastic fluctuations (so that magnitude of those fluctuations even could be considered as a breakdown when maximum chaotic behaviour would be expected), in fact did not cause the enhanced complex plankton behaviour.

One mesocosm (C) demonstrated an example of chaotic dynamics of three biotic components from two trophic levels: heterotrophic consumers (protists *Arcella* sp., and rotifers *Lecane* sp.), and decomposers – the bacteria (Fig. 1). Importantly, this mesocosm was characterised by the lowest among the vanishingly small variations of abiotic parameters compared to the other mesocosms (Fig. 1, Supplementary Fig. S1). Thus, each episode of chaos therein hypothetically should propagate through the trophic web since the oscillators coupled together likely increase the potential for incommensurate frequencies^2^ due to space-time correlated interconnections of organisms with numerous nutrient cycles, matter and energy flows^44^. However, this effect was not observed. Neither it was possible to reveal the statistically significant multiple signs of chaotic behaviour in the other three mesocosms (Supplementary Fig. S1 and Table S1).

Meanwhile, it must be taken into account that chaotic behaviour cannot necessarily be detected by the direct analyses of the experimental variables. Testing combinations of the experimental variables instead of analysing them separately may result in a stronger indication of the chaotic dynamics. However, such an attempt with respect to the signal/noise ratio of the dataset and the resulting number of false-positive indications was not made in this study. It would probably require an even higher sampling frequency and should also consider the data about physiological activity, especially of the bacterial component in plankton.

Comparative analysis showed that none of the mesocosms exhibited reliable signs of chaotic dynamics of plankton as a whole; however, complex behaviour was revealed for certain components within one of the two assessed time frames (but never in both). Only one out of 4 experimental mesocosms (A) exhibited at least comparable results for both time frames and for at least 2 biotic components (Table 1). Thus, episodes of chaos in plankton of the abiotically stabilized systems were shown to be an option rather than a must. Remarkably, chaos emerged presumably in the most stable abiotic conditions; however, once appeared, those patterns were either spreading within the trophic level or vanishing due to internal biotic interactions. These results illustrate that chaos may be evident or any trace of it may disappear in an experimental time series due to small variations in the abiotic parameters reflecting the temporal and spatial scales of the respective information which these parameters contain.

## Outlook

Historical and current knowledge demonstrates that nearly every real-world system is a nonlinear dynamical integrity, and while some of those may contain only few interacting parts and follow rather simple rules, still all have strong dependence on the initial conditions^11,45^. In nature, aquatic ecosystems represent the many-component life systems of the highest dynamism due to short generation cycles, high metabolic rates, effective adaptation strategies and rapid evolution of major plankton inhabitants^39–41,46–49^. Unravelling the internal mechanisms that rule nonlinear plankton dynamics in nature requires long-term experimenting^44^, particularly in the conditions of stable external triggers, e.g. irradiance, temperature and salinity known as the apt master factors for plankton communities^50^.

A decade ago, strong arguments were put forward in favour of chaotic behaviour of the species-depleted plankton communities^8^. However, the questions whether or not chaos can be detected in the live systems with stable abiotic parameters and can the complex dynamics of plankton be replicated in the mesocosm experiments remained unclear. We suggested an original experimental design^42^ and developed a novel approach that allowed eliminating the multiple potential sources of uncertainty present in some previous studies. Specifically, we performed a long-term (286 days) replicated experiment in 4 mesocosms with multi-species plankton community in the strictly controlled stable abiotic conditions. This study showed that traits of the chaotic dynamics that are routinely associated with changing external conditions may be adaptive even when the extrinsic environment is spatially and temporally homogeneous (Fig. 1). These results are in accordance with the earlier findings of Fussmann and Heber^2^.

Interestingly, temperature and irradiance data sometimes also tended to expose pseudo-chaotic patterns despite the established fact that those parameters were kept constant and instrumentally controlled throughout the experiment^42^. These trends should be considered as ‘false-chaotic’ since they arose from the exceptional sensitivity of the Lyapunov exponent analysis, as demonstrated by our quality indication system and the ICI. This outcome supports the viewpoint that nonlinear systems are difficult to resolve analytically because they cannot be split into constituent parts, solved individually, and then recombined to get a final solution^11^. Therefore, similarly to the 0–1 indicator for resolution of partially predictable chaos from strong chaos and laminar flow in physics^51^, our results highlight the importance of critical follow-up assessment of the Ly-exponent calculations and their interpretation by means of the newly developed Integral Chaos Indicator. These findings also provided experimental evidence to the assumption that even well-controlled biotic systems without external triggers may be “subject to stochastic perturbations” *sensu* Massoud and co-authors^44^.

Unpredictability of chaos hinders forecasting the future of global systems that may eventually become unknowable with any precision because even small interventions may cause unexpected environmental, economic or societal consequences as minor effects can compound nonlinearly over time^11^. Therefore, like innovative Toyo Ito’s architecture and city development in modern Tokyo are akin to ‘finding order in chaos’ and thus become influential^4^, our improved assessment of chaos in nature has imperishable importance. This assessment is crucial since complex dynamics impacts biodiversity nonlinearly^1,19^, resolves multidimensional essence of stability^1,3^ and, therefore, has to be considered in the advanced environmental management^24^.

Our approach to detection and verification of chaotic behaviour opens new windows for Translational Ecology^41^ by showing that chaos is an inherent feature of life systems which ensures their continuity. This new knowledge backs up a predictive understanding of complex systems thus facilitating the essential human life activities. The results of this study will have wider implications in forecasting behaviour of a variety of global life systems, including human society, since we proved experimentally that a lack of external triggers is a poor guarantee that chaos will not be triggered internally and emerge spontaneously, propagating through any level of systems’ hierarchy.

## Conclusions

The authentic long-term experimental study of chaos in natural plankton communities was run in four originally designed replicated mesocosms under the uniquely stable, instrumentally controlled abiotic conditions during 286 days. Plankton in all experimental compartments consisted of bacteria, unicellular picocyanobacteria, colony-forming cyanobacteria, flagellates, heliozoans, testate amoebae, ciliates, rotifers, cladocerans and copepods. These assemblages were representing four trophic levels: primary producers, primary and secondary consumers, and decomposers. A clear distinction between the initial acclimation phase and the subsequent plankton succession was revealed and analyzed statistically. Our innovative results supported both research hypotheses that were set forward and tested in this study and allowed concluding the following:

1. The unconventional natural phenomenon of the “paradox of chaos” was revealed; this implies that biotic interactions act as both triggers and drivers for plankton dynamics in the systems lacking abiotic stressors.
2. Chaos in plankton was registered as episodes at different trophic levels; however, the signs of chaos vanished unpredictably or were substituted by complex behaviour of other candidates when longer time series were considered.
3. We developed the Integral Chaos Indicator – a novel tool for assessing complex behaviour and validating the results of the Lyapunov exponent analysis which is known to be sensitive to variations of the experimental parameters.

## Methods

### Mesocosm setup

A set of four identical 120-L plastic barrels formed the mesocosm compartments labelled A, B, C and D for plankton incubation. All mesocosms were inoculated with a 1:4 mix of water: 1 part was obtained from a previously constructed mesocosm setup (started in 2008 with water initially collected in a coastal lagoon of the Baltic Sea, the Darß-Zingst Bodden, site Zingster Strom), and 3 parts of natural brackish water were taken in July 2010 from the same site. Plankton in the initial inoculum in all experimental compartments consisted of bacteria, unicellular picocyanobacteria, colony-forming cyanobacteria, flagellates, heliozoans, testate amoebae, ciliates, rotifers, cladocerans and copepods. These assemblages were representing four trophic levels: primary producers, primary and secondary consumers, and decomposers^42^. As shown recently by molecular data, most *Microcystis*-like and *Aphanothece*-like morphospecies that originated from the Darss-Zingst Bodden are currently attributed to alpha-picocyanobacteria, being genetically most close to *Cyanobium* which taxonomy is largely unresolved^43^.

Each mesocosm was surrounded by a water jacket (300 L) for temperature control. All water jackets were interconnected by an immersion pump (AquaMedic, OR3500) to form a common closed thermostabilizing system controlled by a cryostat (AquaMedic, SK2). The system provided identical water temperature (20 °C) inside all mesocosms throughout the entire 1 year-long experiment: from August 2010 through September 2011^42^.

To prevent formation of abiotic gradients and stratification, each mesocosm was equipped with a stirrer (MFA/Como Drills; 919D Series connected to a 9.5 cm plastic propeller) placed in the middle of the water column. Low rotating speed (72 rounds min^−1^) and specific stirring regime (5 min on and 5 min off) resulted in smooth water circulation.

The mesocosms were covered with transparent acryl plates in order to diminish evaporation and heat losses. Illumination under a 12 h/12 h dark/light cycle was provided by fluorescent lamps (OSRAM DuluxStar 23 W/825) allowing for up to 135–140 µMol photons m^−2^ s^−1^ at the water surface.

Abiotic parameters (light extinction, irradiance, temperature, pH and salinity), concentration of nutrients (orthophosphate, nitrate, nitrite, ammonium, total nitrogen and total phosphorus in seston), the bacteria, phyto- and zooplankton were sampled twice weekly, evaluated and analyzed using standard laboratory techniques. Further details of the mesocosms design, sampling procedures, species identification and measurement techniques are provided by Schubert and co-authors^42^.

### Statistical analyses

Statistical analyses of the kinetics of abiotic parameters and plankton populations were performed for (1) the stationary window (i.e. the period of time after the initial acclimation of the community has taken place) and (2) the entire dataset by means of the correlation analysis, Spearman rank correlation analysis, non-metric multi-dimensional scaling (MDS) using a Bray-Curtis dissimilarity matrix, and the principal component analysis (PCA). The similarity/dissimilarity between groups of samples was tested by the analysis of similarities (ANOSIM). Similarity percentage analysis (SIMPER) was used to examine the contribution of each set of samples to the average dissimilarity between groups of samples. For all statistical tests, analyses and visualization of the results we used the program package PRIMER V6 (Primer-E Ltd, Plymouth, UK).

### Lyapunov exponent analysis

Dynamics of plankton and the environmental parameters were evaluated by calculation of the Lyapunov exponent (Ly), a measure of convergence/divergence of nearby trajectories that was performed for all available data from 4 mesocosms sampled in 2010–2011. In total, eleven pelagic components were assessed: 5 biotic groups and 6 abiotic parameters. Abundances of 3 zooplankton groups: testate amoebas, rotifers and “other zooplankton”; 2 microplankton compartments: the bacteria and picophytoplankton; and abiotic characteristics: soluble reactive phosphorus (SRP), light extinction, irradiance, temperature, pH, and salinity – were measured and analyzed on an equidistant grid with a time step of 3.5 days.

The final dataset covered 286 days and consisted of *n* = 97 observations of each species’ population counts and *n* = 130 measurements of each abiotic parameter. Data were fourth-root power transformed as described by Benincà with co-authors^8^, for homogenising the variance of all counts; none of the trends were excluded from the analyses.

For test purposes, additional five time series were generated artificially: (1) a sinus function describing a purely deterministic dynamics with fluctuations of the parameter; (2) time series as in (1) but with addition of a random component; (3) a uniform time series with random numbers; (4) a size-ordered time series based on the original data on *Arcella* sp. from mesocosm A, with monotonically increasing values; and (5) a time series consisting of about 100 points from two cycles (6 loops on the two wings, flipping twice from one to the other) of the chaotic Lorenz system mimicking pseudo-deterministic behaviour.

All time series were analyzed using the “lyap_r” function of the Tisean V3.0.1 package^52,53^. Negative Lyapunov exponent values (Ly-values) indicated that the nearby trajectories converged, which was considered as a characteristic of stable equilibria and periodic cycles in the population. Conversely, positive Ly-values represented divergence of the trajectories indicating the complex (chaotic) behaviour.

## Supplementary information

Supplementary Information.

## Data Availability

All data generated and/or analyzed during this study are available from the corresponding author on reasonable request.

## Code availability

The authors confirm that should the manuscript be accepted, the data supporting the results will be published online as Supplementary Information.
