# Supplementary material for: Chaos theory discloses triggers and drivers of plankton dynamics in stable environment
Source: Sci Rep. 2019 Dec 30;9:20351. doi: 10.1038/s41598-019-56851-8 (PMC6937249; doi:10.1038/s41598-019-56851-8)
Supplement: Supplementary file 1 — Supplementary Information. [file 41598_2019_56851_MOESM1_ESM.pdf]

Supplementary Information

Manuscript title:

Chaos theory discloses triggers and drivers of plankton dynamics in stable environment

Authors:

Irena V. Telesh*, Hendrik Schubert, Klaus D. Joehnk, Reinhard Heerkloss, Rhena Schumann, Martin Feike, Arne Schoor & Sergei O. Skarlato

Supplementary Figures

Supplementary Fig. S1 | Abiotic stability in the experimental mesocosms. Box-Whisker plots of irradiance (**a**), water temperature (**b**), and salinity (**c**) in the mesocosms during two experiments: in 2010-2011 (A1-D1, described in this study) and 2012-2013 (A2-D2)[41]. Shown are minimum, mean and maximum values, 25% and 75% quartiles. Y-Axis stretched.

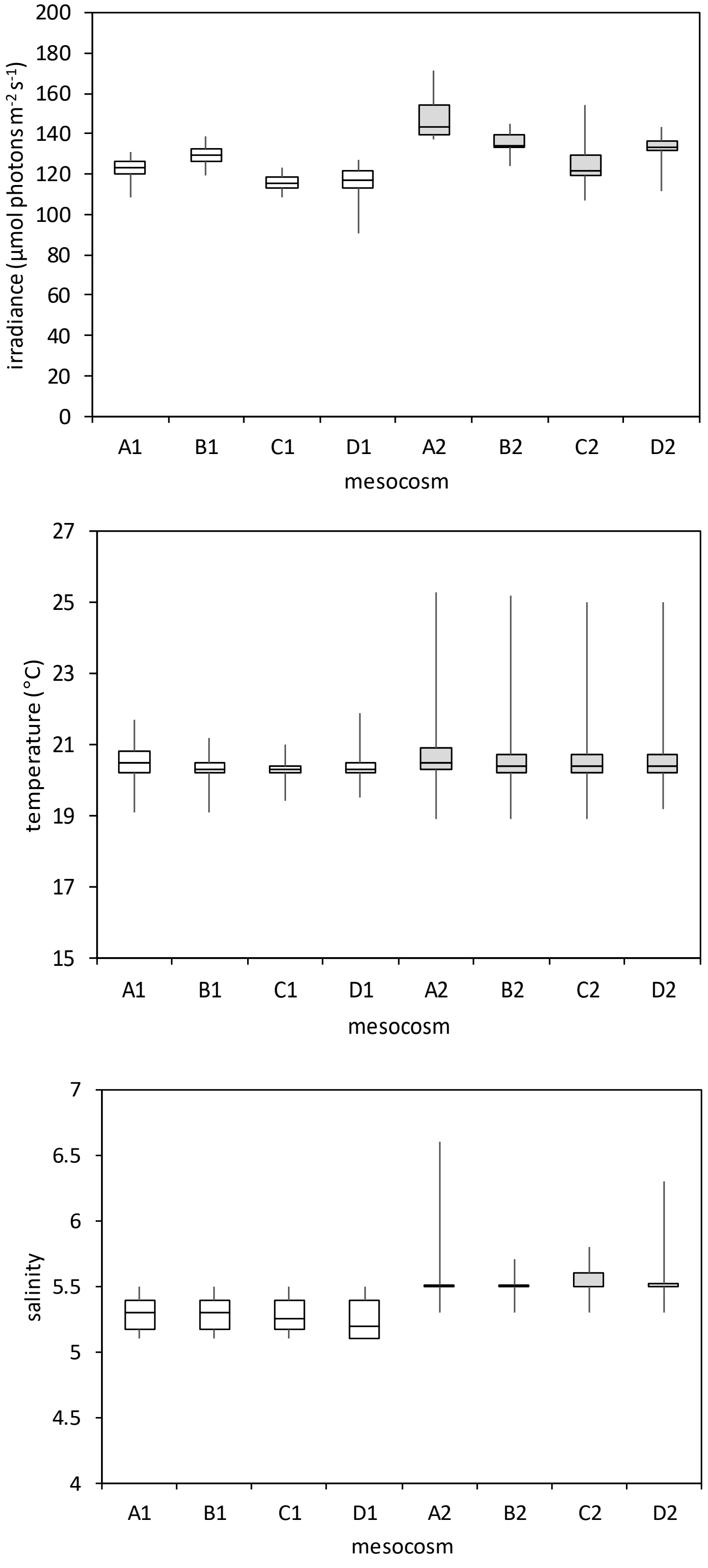

Supplementary Fig. S2 | Dynamics of microplankton and nutrients in the mesocosms. A, B, C, D – mesocosms. **a**, Density of bacteria (10^6 cells mL^{-1}), approximated by the exponential equations (R^2: 0.44 – 0.56). **b**, Abundance of picocyanobacteria (10^6 cells mL^{-1}). **c,** Numbers of '*Microcystis*' * (cells $colony^{-1}$, used to estimate biomass of colonies counted in total phytoplankton samples in the sedimentation chamber), approximated by the exponential logarithmic trend lines (R^2: 0.32 – 0.61). **d,** Numbers of '*Aphanothece*' * (cells $colony^{-1}$; same as for '*Microcystis*'). **e**, Concentration of PO_4^{3-} (µMol L^{-1}). **f**, DIN concentration (µMol L^{-1}). **g**, DIN/DIP ratio (M/M, log_{10}-scale). **h**–**m**, Population density (ind. L^{-1}) of *Arcella* sp. (**h**), *Lecane* sp. (**i**), *Colurella* sp. (**j**), *Filinia longiseta* (**k**), *Brachionus quadridentatus* (**l**), and *Keratella cochlearis* (**m**). * – as shown recently by molecular data, most *Microcystis*-like and *Aphanothece*-like morphospecies that originated from the Darss-Zingst Bodden are currently attributed to alpha-picocyanobacteria , being genetically most close to *Cyanobium* which taxonomy is largely unresolved[43].

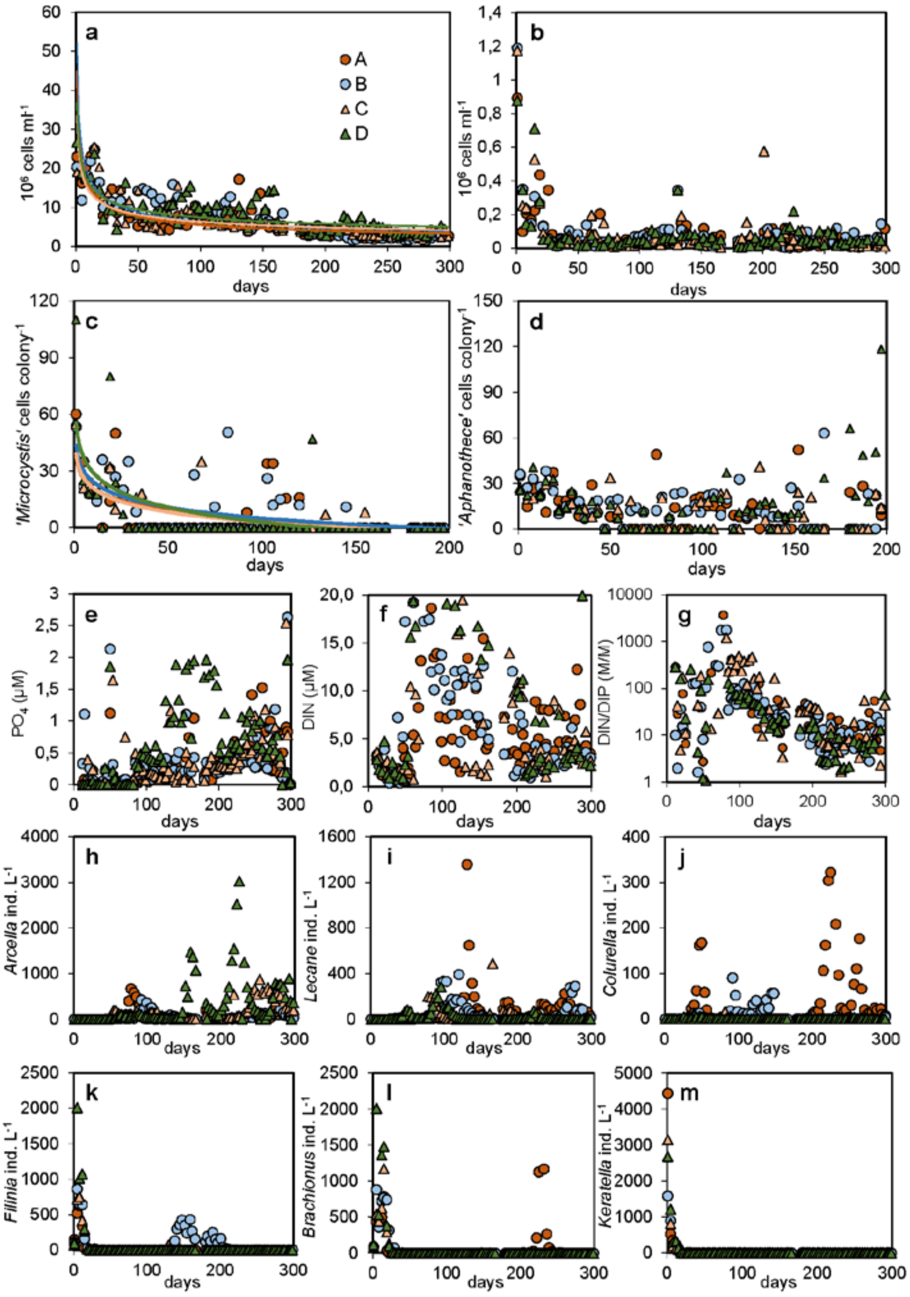

Supplementary Fig. S3 | Zooplankton abundance and biomass. Dynamics of population density (**a–e**; ind. L^{-1}) of crustaceans *Eurytemora affinis* (**a**), *Alona* sp. (**b**), *Acartia tonsa* (**c**), cyclopoid copepods (**d**), other zooplankters (**e**), and the overall zooplankton biomass (**f–i**; µg L^{-1}) in the mesocosms A, B, C, and D.

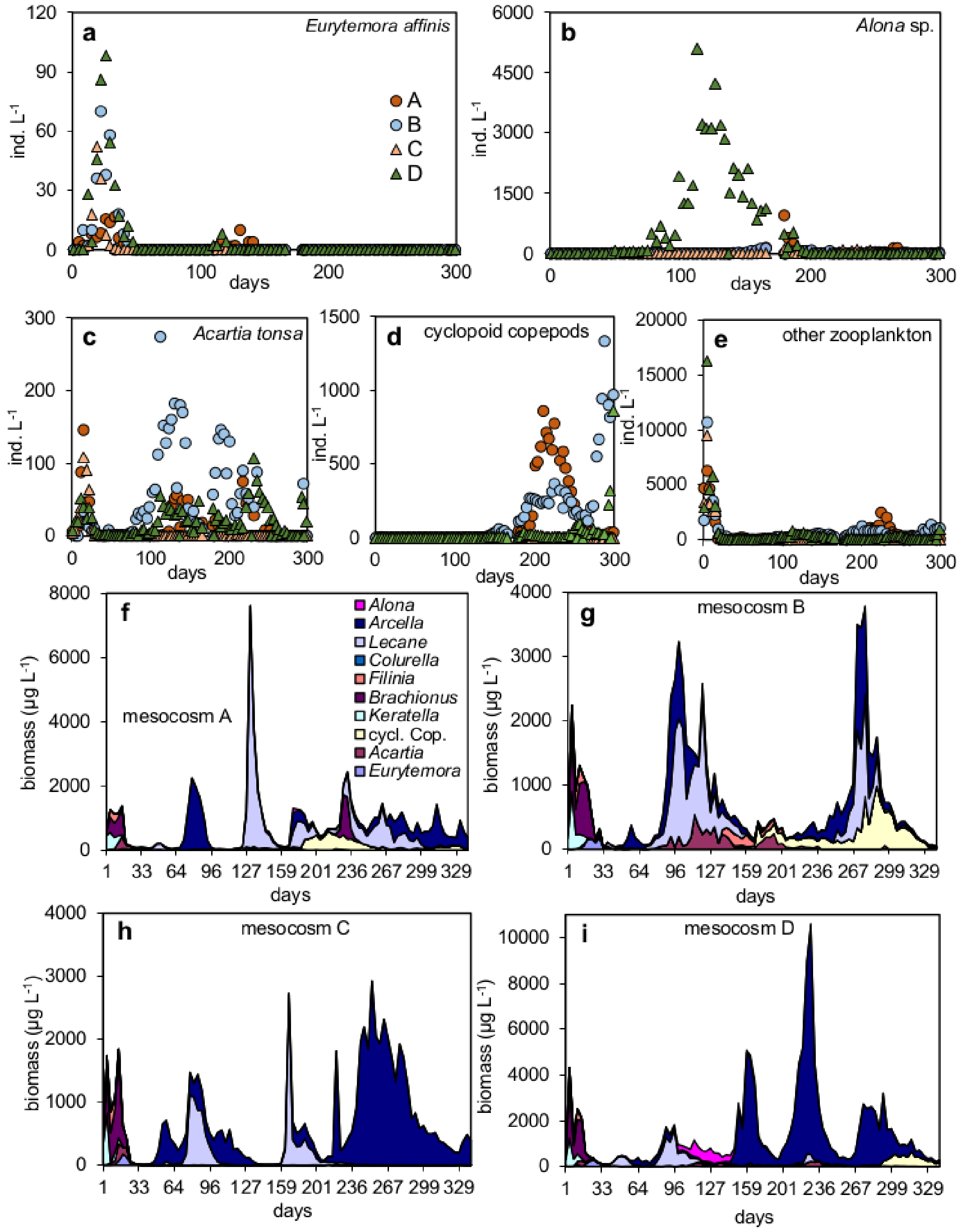

Supplementary Fig. S4 | Lyapunov exponent analysis for the stationary window dataset and its assessment by the Quality Indicators. Each graph includes the points calculated by Tisean v3.0.1 (empty dots), a combined fit for the linear increase and plateau (red line), a significance indicator (+, 0, -) for the slope and the plateau, a color dot indicating the relevance of the calculated Ly-values (green – positive test for both quality indicators (QI1 and QI2), yellow – positive test for just one of the indicators, red – negative tests for both indicators), and (in some cases) horizontal lines that help discriminating the size of the wiggles of the plateau. A, D, C, and D – mesocosms. Abbreviations: arc – *Arcella* sp., lec – *Lecane* sp., other – other zooplankton, bac – bacteria, pic – picophytoplankton, SRP – soluble reactive phosphorus, ext – light extinction, ph – pH, tem – temperature, irr – irradiance, sal – salinity; Det – test 1, deterministic; Det+Rnd – test 2, deterministic + random; Rnd – test 3, random; Ord – test 4, ordinated Aarc; Lor – test 5, Lorenz attractor.

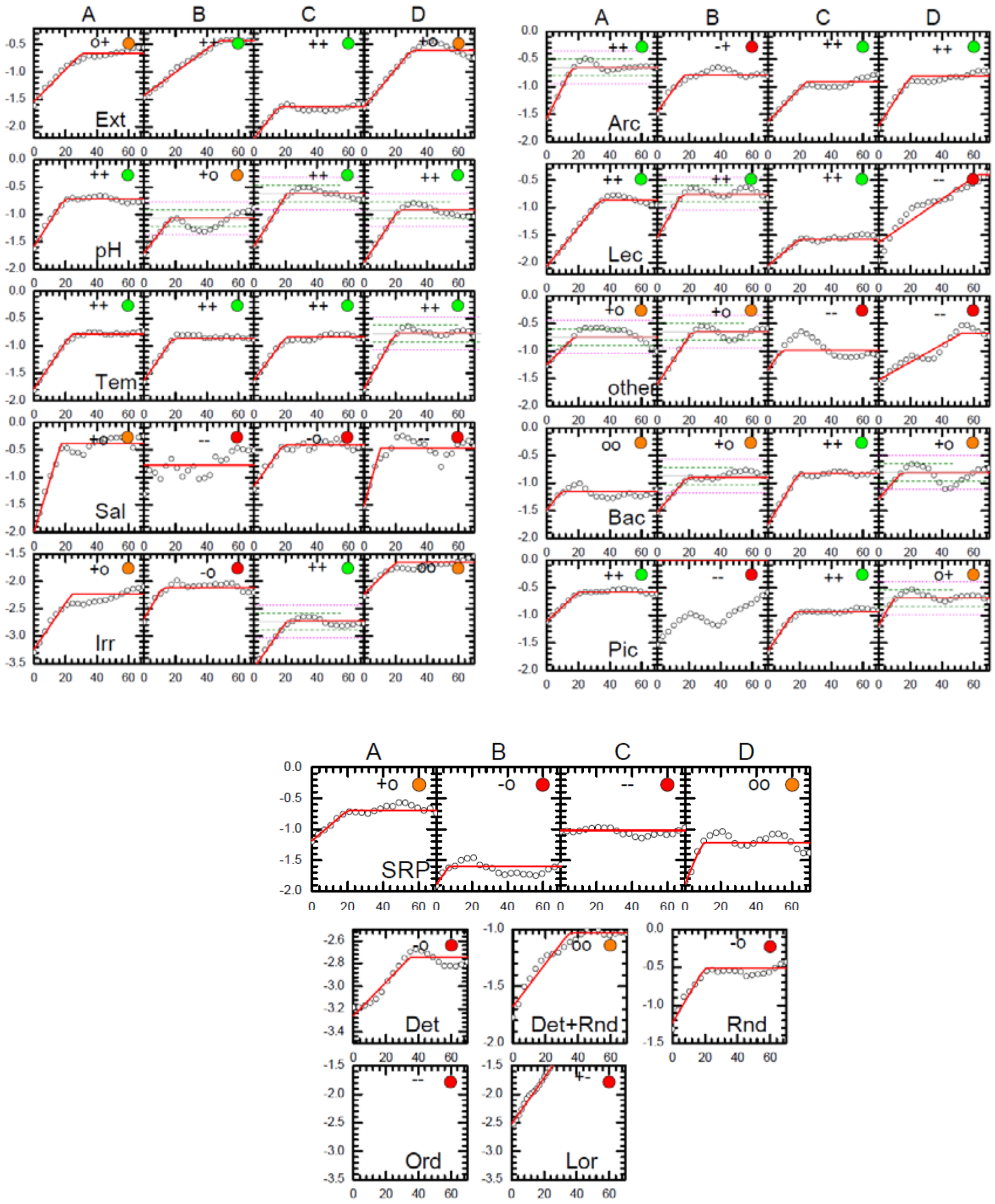

Supplementary Fig. S5 | Lyapunov exponent analysis for all time-series and all data and its assessment by the Quality Indicators. Indication and abbreviations as in Supplementary Fig. S2.

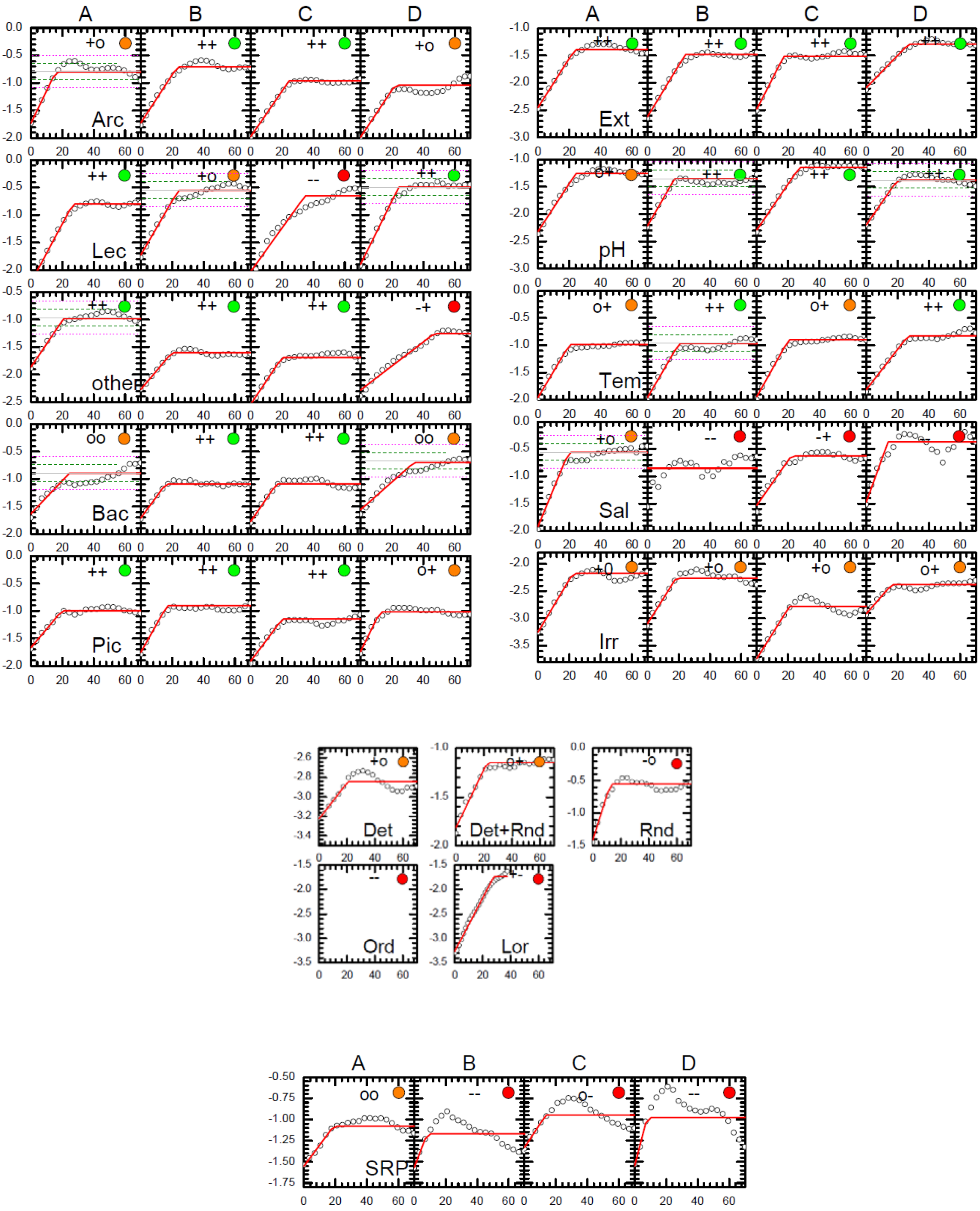

Supplementary Fig. S6 | Histogram and probabilities of fitted slope values (Lyapunov values) for 100 randomly shuffled time-series of the stationary window dataset. The arrow indicates the Ly-value estimated from the original time series (which is not necessarily the result of a good fit).

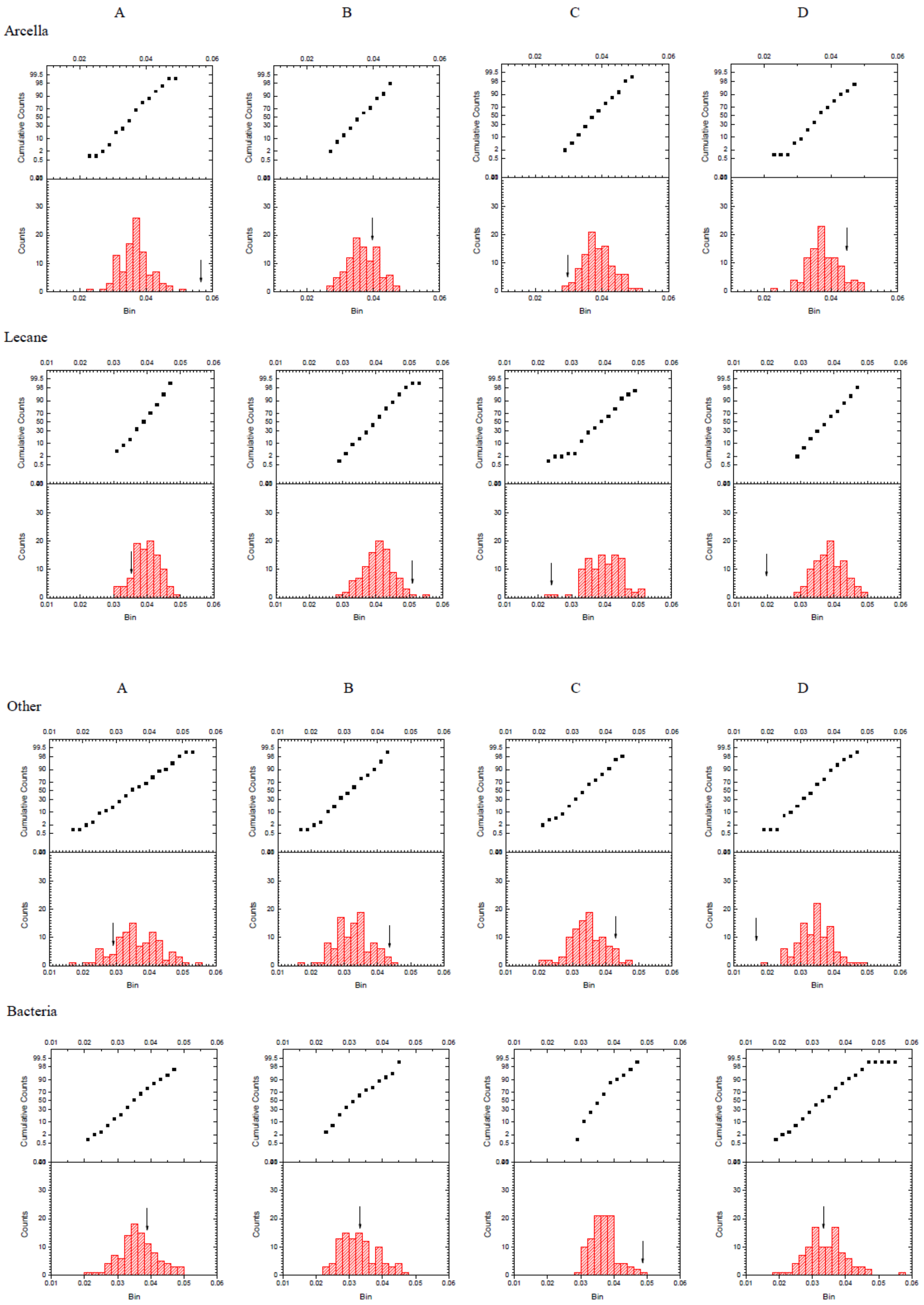

A B C D

Picophytoplankton

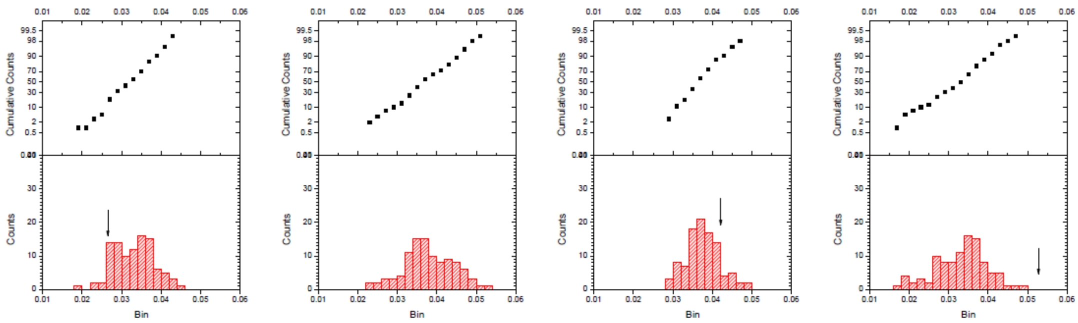

SRP

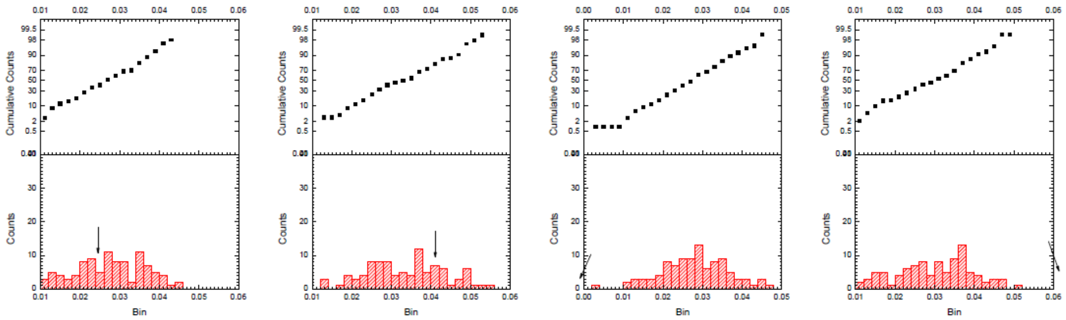

A B C D

Light extinction

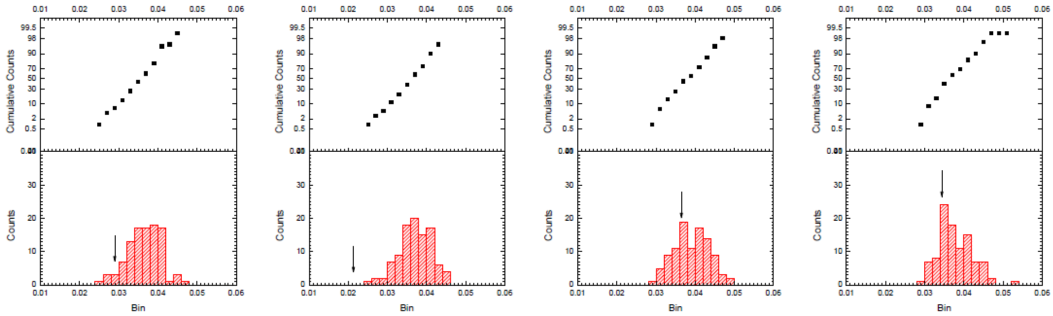

pH

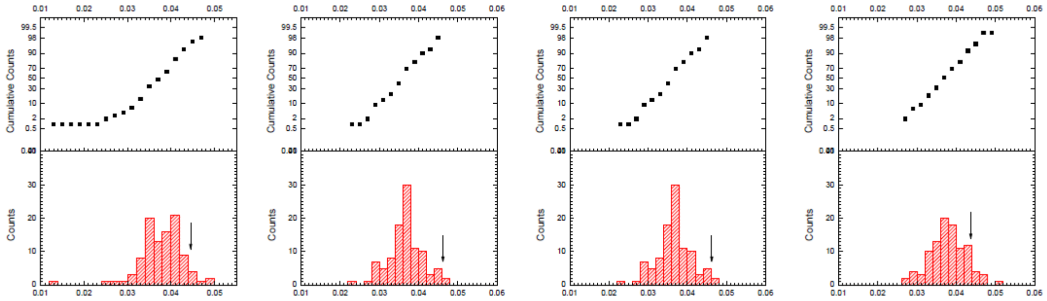

A B C D

Salinity

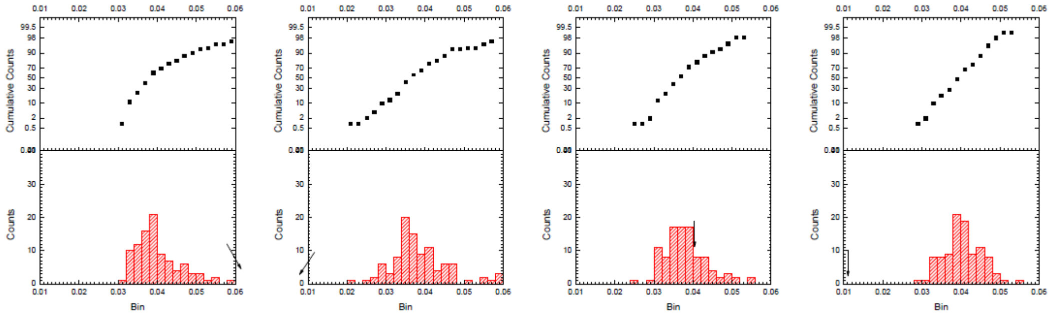

A B C D

Temperature

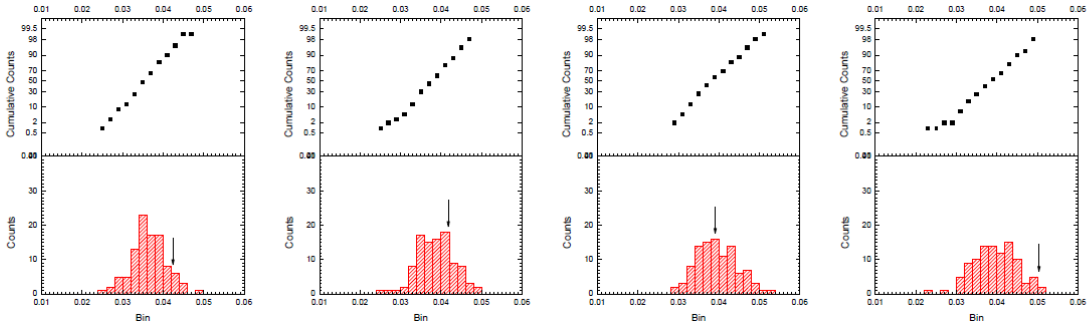

Irradiance

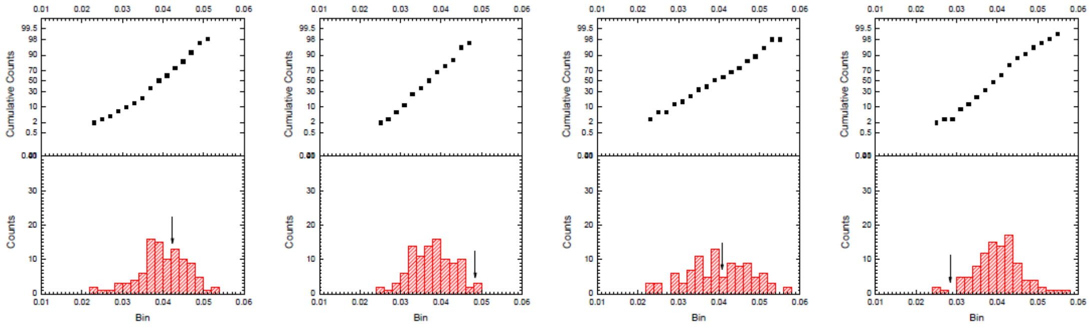

Test cases

Deterministic (sinus) | Deterministic + random noise | Random noise | Ordered time series

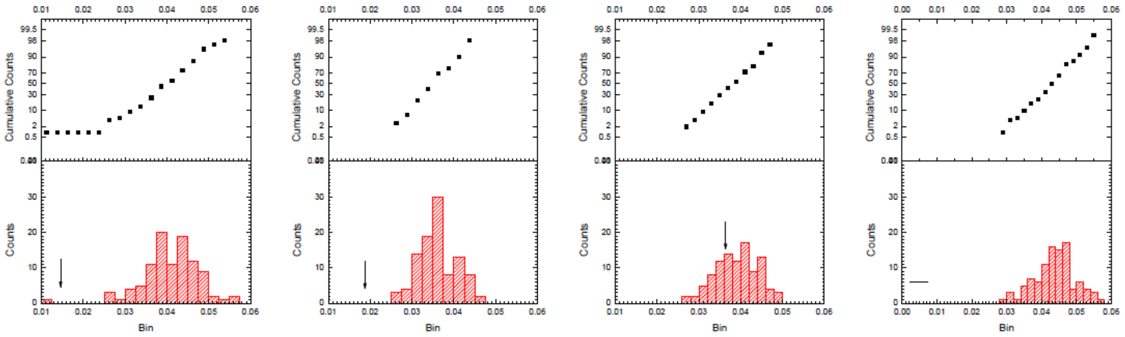

Deterministic (2 cycles on Lorenz attractor)

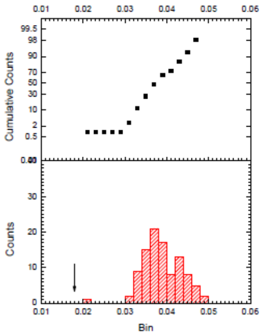

Supplementary Tables

Supplementary Table S1 | Results of Spearman Rank Correlation Analysis. DIN and PO_4^{3-} concentration dynamics and similarity of these patterns in the mesocosms A–D; *p* – Pearson correlation coefficient, P – probability (asymptotic significance).

Mesocosms	DIN		PO_4^{3-}	
	***p* (68 values)**	**P**	***p* (57 values)**	**P**
A/B	0.447	0.553	0.531	0.469
A/C	0.490	0.510	0.556	0.444
A/D	0.603	0.396	**0.291**	0.709
B/C	0.429	0.571	0.533	0.467
B/D	0.684	0.316	**0.396**	0.604
C/D	0.599	0.401	**-0.067**	1.067

Supplementary Table S2 | Details of the PCA results as shown in Fig. 3. Abbreviations: Pic - picoplankton abundance (10^6 Ind. ml^{-1}); Bac - bacteria abundance (10^6 Ind. ml^{-1}); Cbc - cyanobacterial colonies (mL^{-1}); Aph - *Aphanothece* sp. colonies (mL^{-1}); Mic - *Microcystis* sp. colonies (mL^{-1}); Gom - *Gomphosphaeria* sp. colonies (mL^{-1}); EUR - *Eurytemora affinis* (Ind L^{-1}); ACA - *Acartia tonsa* (Ind L^{-1}); CYC - cyclopoid Copepods (Ind L^{-1}); KEC - *Keratella cochlearis* (Ind L^{-1}); BQU - *Brachionus quadridentatus* (Ind L^{-1}); FIL - *Filinia longiseta* (Ind L^{-1}); COL - *Colurella* sp. abunance (Ind L^{-1}); LEC - *Lecane* sp. (Ind L^{-1}); ARC - *Arcella* sp. (Ind L^{-1}); ALO - *Alona* sp. (Ind L^{-1}); OTH - other zooplankton species abundance (Ind L^{-1}); ZOO - total zooplankton biomass (FW).

Panel a

Eigenvalues

PC	Eigenvalues	%Variation	Cum.%Variation
1	2,12	30,2	30,2
2	1,36	19,4	49,7
3	1,23	17,6	67,3
4	0,905	12,9	80,2
5	0,627	9,0	89,2

Eigenvectors

(Coefficients in the linear combinations of variables making up PC's)

Variable	PC1	PC2	PC3	PC4	PC5
DIP	0,142	0,691	-0,128	0,227	-0,407
DIN	-0,334	0,122	-0,609	-0,181	-0,455
Pic	-0,204	0,097	0,648	-0,498	-0,476
Bac	-0,606	-0,013	0,145	-0,026	0,100
Cbc	-0,316	0,164	0,336	0,755	-0,046

pH	0,574	0,205	0,244	-0,041	-0,069
ZOO	-0,170	0,655	-0,005	-0,309	0,620

Panel b

Eigenvalues

PC	Eigenvalues	%Variation	Cum.%Variation
1	199	36,4	36,4
2	145	26,5	62,8
3	77,1	14,1	77,0
4	54,7	10,0	87,0
5	27	4,9	91,9

Eigenvectors

(Coefficients in the linear combinations of variables making up PC's)

Variable	PC1	PC2	PC3	PC4	PC5
DIP	0,001	-0,010	-0,017	-0,002	-0,002
DIN	-0,020	-0,062	-0,001	-0,018	-0,092
Pic	0,003	0,000	0,002	-0,004	0,002
Bac	0,008	-0,010	0,044	-0,046	-0,017
Aph	0,013	-0,010	0,090	-0,087	-0,010
Mic	0,024	0,012	0,069	-0,068	0,022
Gom	-0,007	0,000	-0,018	-0,010	0,010
EUR	0,026	0,012	0,078	-0,058	0,029
ACA	0,120	-0,032	-0,016	-0,137	-0,171
CYC	0,293	0,185	-0,423	0,675	0,181
KEC	0,092	0,028	0,060	-0,172	0,058
BQU	0,312	0,115	0,155	-0,417	0,137
FIL	0,191	0,042	0,103	-0,254	0,027
COL	0,047	0,044	-0,004	0,063	-0,199
LEC	0,019	-0,016	-0,091	0,050	-0,923
ARC	-0,217	-0,061	-0,852	-0,457	0,061
ALO	0,150	-0,968	-0,007	0,095	0,066
pH	0,000	0,001	-0,002	0,002	0,002
OTH	0,827	0,038	-0,163	-0,121	-0,072

Supplementary Table S3 | Results of PCA performed with a dataset excluding the first 50 days of the experiment (the 'acclimation phase'). Abbreviations as in Supplementary Table S2.

Eigenvalues

PC	Eigenvalues	%Variation	Cum.%Variation
1	2,22	31,8	31,8
2	1,62	23,1	54,9
3	1,26	18,0	72,9
4	0,709	10,1	83,0
5	0,52	7,4	90,5

Eigenvectors

(Coefficients in the linear combinations of variables making up PC's)

Variable	PC1	PC2	PC3	PC4	PC5
DIP (µM)	0,150	-0,356	0,588	0,536	0,411
DIN (µM)	-0,223	0,358	0,604	-0,191	-0,412
Pic	-0,404	-0,300	-0,250	0,569	-0,579
Bac	-0,592	0,103	-0,048	0,157	0,389
pH	0,481	-0,442	-0,067	-0,087	-0,256
ZOO	-0,265	-0,461	0,431	-0,330	-0,202

Supplementary Table S4 | Results of tests for differences between phases and mesocosms by means of a nested two-way approach.

TESTS FOR DIFFERENCES BETWEEN PHASES across all mesocosms
Global Test
Sample statistic (Global R): 0,388
Significance level of sample statistic: 0,1%
Number of permutations: 999 (Random sample from a large number)
Number of permuted statistics greater than or equal to Global R: 0

TESTS FOR DIFFERENCES BETWEEN mesocosm GROUPS using phase groups as samples
Global Test
Sample statistic (Global R): 0,132
Significance level of sample statistic: 8,1%
Number of permutations: 999 (Random sample from 488864376)
Number of permuted statistics greater than or equal to Global R: 80

Pairwise Tests

Groups	R Statistic	Significance Level %	Possible Permutations	Actual Permutations	Number >= Observed
C, A	0,148	16,7	126	126	21
C, B	0,348	7,1	126	126	9

C, D	0,24	4	126	126	5
A, B	0,068	22,2	126	126	28
A, D	-0,048	60,3	126	126	76
B, D	0,02	34,1	126	126	43

TESTS FOR DIFFERENCES BETWEEN mesocosm GROUPS across all phase groups
Global Test
Sample statistic (Global R): 0,428
Significance level of sample statistic: 0,1%
Number of permutations: 999 (Random sample from a large number)
Number of permuted statistics greater than or equal to Global R: 0

TESTS FOR DIFFERENCES BETWEEN phase B GROUPS using mesocosm groups as samples
Global Test
Sample statistic (Global R): 0,259
Significance level of sample statistic: 1,4%
Number of permutations: 999 (Random sample from a large number)
Number of permuted statistics greater than or equal to Global R: 13

Pairwise Tests

Groups	R Statistic	Significance Level %	Possible Permutations	Actual Permutations	Number >= Observed
B1, B2	0,344	2,9	35	35	1
B1, B3	0,219	11,4	35	35	4
B1, B4	0,271	8,6	35	35	3
B1, B5	0,656	2,9	35	35	1
B2, B3	0,333	5,7	35	35	2
B2, B4	0,344	11,4	35	35	4
B2, B5	0,781	2,9	35	35	1
B3, B4	-0,052	45,7	35	35	16
B3, B5	0,083	17,1	35	35	6
B4, B5	-0,146	82,9	35	35	29
